# Phosphatidic Acid Serves as an Early Signaling Molecule Involved in Membrane Lipid Metabolism to Alleviate Postharvest Chilling Injury in Peach Fruit

**DOI:** 10.3390/foods15162810

**Published:** 2026-08-12

**Authors:** Yun Zhang, Fengyuan Xie, Ziyi Wang, Yonghua Zheng, Liangyi Zhao, Peng Jin

**Affiliations:** College of Food Science and Technology, Nanjing Agricultural University, Nanjing 210095, China; zhang-yun@stu.njau.edu.cn (Y.Z.); 2023108084@stu.njau.edu.cn (F.X.); wangziyi1028@stu.njau.edu.cn (Z.W.); zhengyh@njau.edu.cn (Y.Z.)

**Keywords:** peach fruit, chilling injury, lipid metabolomics, phosphatidic acid, phospholipase D

## Abstract

Peach fruit develops chilling injury (CI) during cold storage, yet the symptoms are milder at 0 °C than at 5 °C; however, the underlying mechanism remains unclear. This study demonstrated that peach fruit stored at 0 °C maintains higher levels of structural phospholipids and membrane lipid unsaturation, along with lower activities of lipase and lipoxygenase (LOX), thereby alleviating membrane damage. Using lipidomics, we identified phosphatidic acid (PA) as a lipid closely associated with CI characteristics and revealed that it plays a signaling role at the early stage of storage at 0 °C, while its reduced levels at the later stage alleviate membrane damage, thereby participating in the regulation of CI in peach fruit. Moreover, at 0 °C, Phospholipase D (PLD) activity exhibited an initial increase followed by a decrease, which was consistent with the overall changes in PA content. During storage, PLD activity and PA content at 0 °C were significantly lower than those at 5 °C and 15 °C. These findings highlight the critical role of PA in temperature-dependent regulation and provide potential molecular targets for precise management of CI in the postharvest cold chain of peach fruit.

## 1. Introduction

Peach (*Prunus persica* (L.) Batsch) is prized for its vibrant coloration, distinctive flavor, and high nutritional value, which contribute to its considerable economic importance and widespread consumer preference [1,2]. As a typical climacteric fruit, peach matures during the hot summer months, and following harvest, it exhibits elevated respiratory activity and metabolic rates, rendering it highly susceptible to rapid senescence and decay. Consequently, low-temperature storage is commonly employed in commercial practice to extend postharvest life. However, peaches are also highly cold-sensitive and prone to chilling injury (CI) under low-temperature conditions [3]. The severity of CI symptoms is closely associated with fluctuations in storage temperature, leading to substantial economic losses in the peach industry [4]. Notably, CI symptoms develop more rapidly and severely at 5 °C than at 0 °C [5]. Research has shown that in ‘Okubo’ peach, CI symptoms appear after 15 d of storage at 5 °C, whereas only slight symptoms are observed after 30 d at 0 °C, characterized by reduced softening capacity, decreased juice yield, and increased relative electrolyte leakage (REL) [6]. In chilling-sensitive peach varieties stored at 0 °C, the CI index at 28 d is approximately 60% lower than that at 5 °C [7]. Furthermore, studies have demonstrated that peach fruit stored at 0 °C exhibits higher membrane lipid unsaturation and membrane fluidity, along with elevated expression of ω-3 fatty acid desaturase genes and higher linolenic acid (C18:3) levels compared to those stored at 5 °C, which contributes to enhanced low-temperature tolerance [8]. The key to this phenomenon may be closely related to the difference in the activation patterns of cellular signaling pathways in fruits stored at different temperatures.

Under cold stress, the cell membrane serves as the primary site for perceiving temperature signals and is also the initial target of injury [9,10]. Low temperatures reduce the fluidity of membrane lipids, inducing a phase transition from a relatively fluid liquid-crystalline state to a more rigid gel phase [11,12]. This phase transition compromises membrane structural integrity, resulting in abnormally increased membrane permeability, leakage of intracellular contents, and disruption of metabolic homeostasis [13,14]. Prolonged exposure to cold stress exacerbates membrane damage, accompanied by phospholipid degradation, a decline in unsaturated fatty acid content within membrane lipids, and the onset of lipid peroxidation [15]. These alterations further increase membrane permeability and promote the accumulation of malondialdehyde (MDA) and other cytotoxic byproducts, ultimately leading to CI. Disruption of the structural and functional integrity of the cell membrane constitutes the fundamental cause of CI. Membrane lipid degradation is primarily orchestrated by membrane lipid-degrading enzymes, including lipoxygenase (LOX) and Phospholipase D (PLD) [16,17].

Early and rapid signal transduction is critical for plants to establish effective adaptive responses to environmental stress [18]. PLD is one of the major enzymes responsible for hydrolyzing membrane lipids to generate phosphatidic acid (PA) and the corresponding polar head groups in plants and is widely involved in various physiological processes, including stress responses, root development, and programmed cell death [19,20]. Under cold stress, phospholipase C (PLC) and PLD in *Arabidopsis thaliana* suspension cells are rapidly activated within minutes, triggering a transient accumulation of PA. As a second messenger, PA initiates downstream cold acclimation responses, highlighting its central role in plant cold signal transduction [21,22,23]. Previous studies have shown that specific molecular species of glycerolipids, including PA, play important roles in plant cold tolerance and can serve as potential biomarkers [24,25]. A recent study found that during the recovery phase of barley roots following long-term cold stress, the levels of two PA molecular species, C34:2 and C36:4, increased significantly, suggesting that acyl editing of PA may be an important mechanism for cold tolerance in barley [26]. PA plays a signaling lipid in plant responses to cold stress, and its accumulation significantly impacts membrane integrity [27]. For instance, excessive accumulation of PA in *Arabidopsis thaliana* has been shown to promote membrane fusion and trigger cell death [28]. In addition, PA can induce the production of hydrogen peroxide (H_2_O_2_) under freezing stress [29]. As a major reactive oxygen species (ROS), H_2_O_2_, when accumulated excessively, leads to membrane lipid peroxidation and degradation [30]. These findings suggest that metabolites derived from PLD-mediated pathways are intricately linked to various stress responses. However, in postharvest fruit such as peach, the role of PLD-PA in response to cold storage and its contribution to CI remain largely unknown.

However, existing studies on the mechanisms underlying temperature-induced differences in CI in peach fruit have primarily focused on membrane lipid metabolism, energy metabolism, and γ-aminobutyric acid metabolism [7,8,31]. The role of PA in temperature-induced differences in CI of peach fruit remains unclear. We hypothesized that the milder CI observed at 0 °C compared to 5 °C is attributable to differential accumulation of PA, which leads to distinct patterns of membrane lipid remodeling and ultimately influences CI development. In order to verify the above hypothesis, this study systematically investigated peaches stored at 0 °C, 5 °C and 15 °C by lipidomics analysis. We dynamically monitored PLD activity, endogenous fatty acid (PA) accumulation, and other membrane lipid components and related enzymatic activities at different storage temperatures. The goal is to clarify whether PA is involved in the regulation of membrane lipid metabolism as an early signaling molecule, thereby mediating the tolerance of peach fruits to temperature-dependent CI, and further elucidate the molecular mechanism of the difference in the severity of CI under low-temperature storage conditions.

## 2. Materials and Methods

### 2.1. Fruit Materials and Sample Preparation

Peach fruit (*Prunus persica* L. Batsch cv. ‘Hujingmilu’) at commercial maturity (firmness: 35 ± 2 N; total soluble solid content: 12 ± 1%) was harvested at the Jiangsu Academy of Agricultural Sciences in Nanjing, China. Upon arrival, the fruits were promptly pre-cooled to remove field heat. A total of 1050 fruits with uniform maturity, free from visible defects and mechanical damage, were selected and randomly divided into three groups of 350 fruits each. The fruits were then stored at 0 °C, 5 °C, and 15 °C, respectively, with a relative humidity of 85–90% for 28 d [7]. At each time point during the early storage period (6, 12, 24, 48, and 72 h), 20 peach fruit samples were collected, and the flesh tissues were immediately frozen in liquid nitrogen and stored at −80 °C for subsequent biochemical and molecular analyses. Thereafter, at weekly intervals (0, 7, 14, 21, and 28 d), 50 peach fruit samples were collected at each time point. Among these, 15 fruits were randomly selected and stored at −80 °C for subsequent analyses; another 15 fruits were used for the measurement of quality-related parameters; and the remaining 20 fruits were transferred to 25 °C with a relative humidity of 85–90% for 3 d to simulate shelf-life conditions. At each shelf-life assessment point (7 + 3 d, 14 + 3 d, 21 + 3 d, and 28 + 3 d), internal browning was documented photographically, and the incidence of decay was recorded.

### 2.2. Measurement of Chilling Injury Index, and Decay Index

Browning index is commonly used as an indicator to evaluate the CI index in peach fruit. The extent of browning was categorized into five scales based on the browned area: 0 represented none, 1 represented 0–25% browning, 2 represented 25–50% browning, 3 represented 50–75% browning, and 4 represented 75–100% browning [32]. The CI index was calculated by ∑[(CI scale) × (number of fruit at that CI scale)]/(4 × total number of fruit).

Decay severity was scored on a four-point scale: 0 represented none, 1 represented 0–25% decayed area, 2 represented 25–50% decayed area, and 3 represented 50–75% decayed area [33]. The decay index was calculated by ∑[(Decay scale) × (Number of fruits at that scale)]/(3 × total number of fruits).

### 2.3. Measurement of Relative Electrolyte Leakage, and Malondialdehyde Content

REL was measured following the protocol described by Zhao et al. [34]. Eighteen flesh discs (2 mm thick, 6 mm in diameter) were collected from six fresh fruits and immersed in distilled water. The initial conductivity (L0) was recorded. After 30 min of immersion, the ion leakage (L1) was measured. Finally, the samples were boiled in distilled water for 15 min, allowed to cool, and the final conductivity (L2) was measured. The ion leakage percentage was calculated as [(L1 − L0)/(L2 − L0)] × 100%. MDA content was determined according to the method of Shen et al. [35], with absorbance readings recorded at 450, 532, and 600 nm. The results were expressed as μmol·kg^−1^ on a fresh weight basis. The MDA content was calculated as C (μmol·L^−1^) = 6.45 × (OD_532_ − OD_600_) − 0.56 × OD_450_.

### 2.4. Measurement of Membrane Phospholipids

PA, phosphatidylcholine (PC), phosphatidylinositol (PI), phosphatidylglycerol (PG), diacylglycerol (DAG), and phosphatidylethanolamine (PE) contents were detected using ELISA kits (Shanghai Yihe Biotechnology Co., Ltd., Shanghai, China) according to the instructions. The PC and PG content were expressed as mmol kg^−1^, PI, PE, and DAG contents were represented as μg kg^−1^, and PA content was represented as nmol kg^−1^ according to fresh weight.

### 2.5. Lipid Metabolism-Related Enzyme Activity Assay

The activities of PLD, PLC, DGK, Lipase, LOX, and fatty acid desaturases (FADs) were detected by ELISA kits (Shanghai Yihe Biotechnology Co., Ltd., Shanghai, China) in accordance with the guidelines provided by the manufacturer. The activities were expressed as U kg^−1^ according to fresh weight.

### 2.6. Lipid Extraction and Analysis

Metabolite extraction and mass spectrometry detection were performed using ultra-high performance liquid chromatography-tandem mass spectrometry (UHPLC-MS, Waters, Milford, MA, USA). Chromatographic separation was achieved using a Waters Acquity UPLC CSH C18 column (1.7 μm, 2.1 mm × 100 mm, Waters, Milford, MA, USA). The mobile phases for both positive and negative ion modes were as follows: mobile phase A consisted of 60% acetonitrile in water containing 10 mM ammonium acetate and 0.1% formic acid; mobile phase B consisted of 90% isopropanol in acetonitrile containing 10 mM ammonium acetate and 0.1% formic acid. The injection volume was 5 μL, and the column temperature was maintained at 55 °C. The electrospray ionization (ESI) source parameters were configured as follows: capillary voltage, 2500 V in positive ion mode and −2000 V in negative ion mode; cone voltage, 30 V; ion source temperature, 120 °C; desolvation gas temperature, 550 °C; cone gas flow rate, 50 L·h^−1^; and desolvation gas flow rate, 900 L·h^−1^. After normalization of raw peak areas to the total peak area, subsequent analyses were conducted. Principal component analysis (PCA) and Spearman correlation analysis were employed to evaluate the reproducibility of both intra-group samples and quality control (QC) samples. Identified compounds were annotated with classification and pathway information from the LipidMaps databases. Using SIMCA software (version 14.1, Sartorius Stedim Data Analytics AB, Umea, Sweden), orthogonal partial least squares (OPLS) regression analysis was performed to examine the associations between differential lipids and CI-related parameters.

### 2.7. Statistical Analysis

Three biological replicates were performed for each sample in the experiment, and all data are presented as mean ± standard error. An independent sample T test was used to determine whether there was a significant difference between different treatments under IBM SPSS Statistics 24 (SPSS Inc., Chicago, IL, USA). When *p* ≤ 0.05, it was considered to have a significant difference.

## 3. Results

### 3.1. Effect of Storage Temperature on Postharvest Quality of Peach Fruit

**Figure 1:**
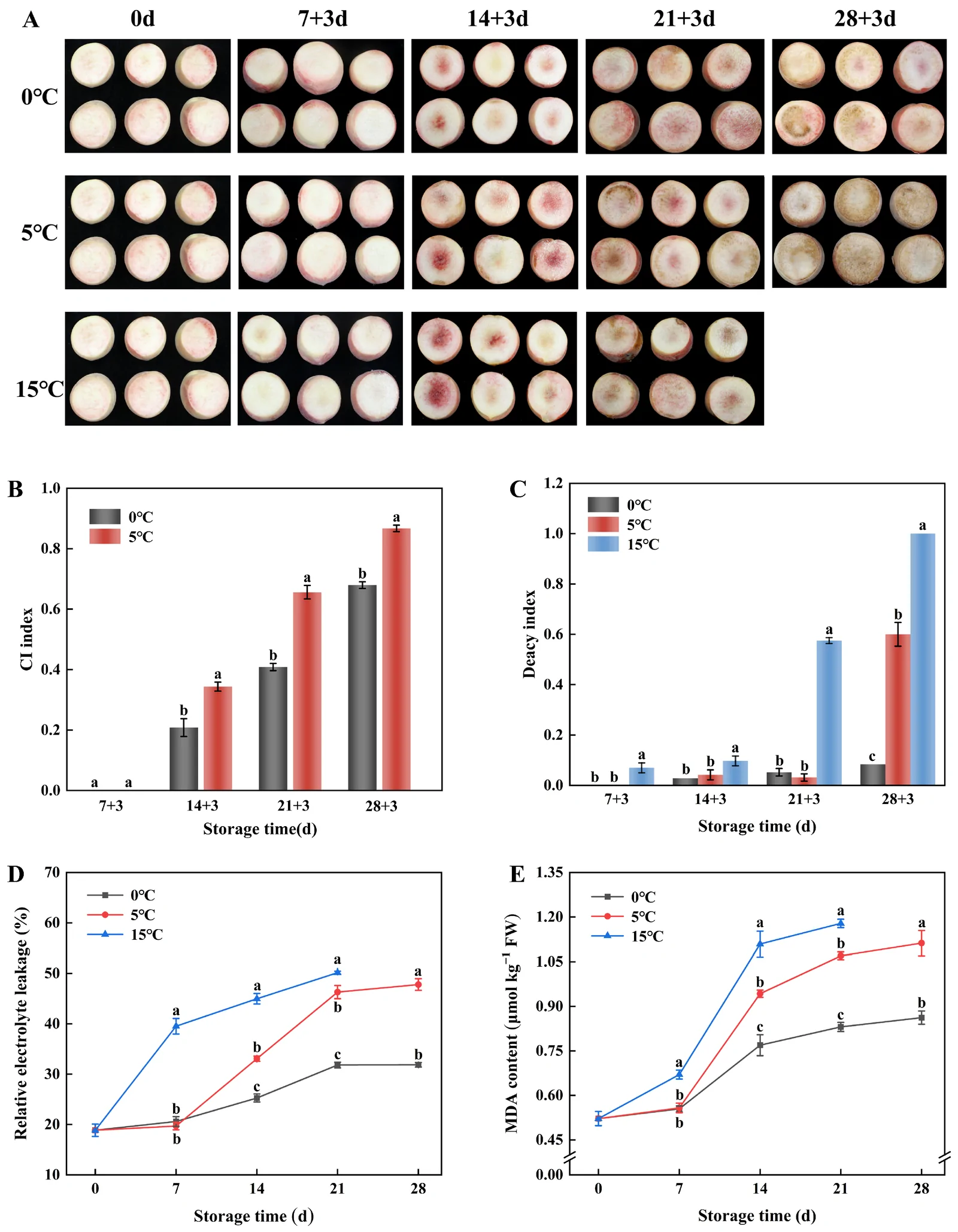
Effects of different temperature treatments on peach fruit. (**A**) Internal appearance of peach fruit after cold storage followed by shelf life. (**B**) CI index. (**C**) Decay index. (**D**) REL. (**E**) MDA content. Data are presented as mean ± standard deviation. Different letters mean statistically significant difference (*p* < 0.05) among peaches stored at the three temperatures on the same day.

Pronounced differences in flesh quality were observed among peach fruits stored at three different temperatures. The onset of CI symptoms was evident after 14 d of refrigerated storage at 0 °C and 5 °C, followed by a 3-day shelf life, with progressive CI developing over time. Notably, CI symptoms were more severe in fruits stored at 5 °C than in those stored at 0 °C during the same period (Figure 1A). By the third week of shelf life, the CI index at 5 °C was 60.71% higher than that at 0 °C (Figure 1B). Fruits stored at 15 °C exhibited no CI; however, the decay index exceeded 0.5 by day 21 and reached 1.0 by day 28. In contrast, peaches stored at 0 °C maintained an extremely low decay index throughout the entire storage and shelf-life period, whereas those stored at 5 °C showed a marked increase in decay index by day 28 (Figure 1C). Overall, peaches stored at 0 °C retained superior quality throughout the experimental period. REL and MDA content exhibited similar upward trends across all three temperature treatments, reaching maximum values at the end of storage. The 15 °C group consistently showed the highest REL and MDA levels throughout the storage duration. After 7 d, both REL and MDA contents in the 5 °C group remained persistently higher than those in the 0 °C group. By day 28, the REL in the 5 °C group was 1.5-fold higher than that in the 0 °C group (Figure 1D,E).

### 3.2. Effects of Low-Temperature Stress on Membrane Phospholipid Composition

**Figure 2:**
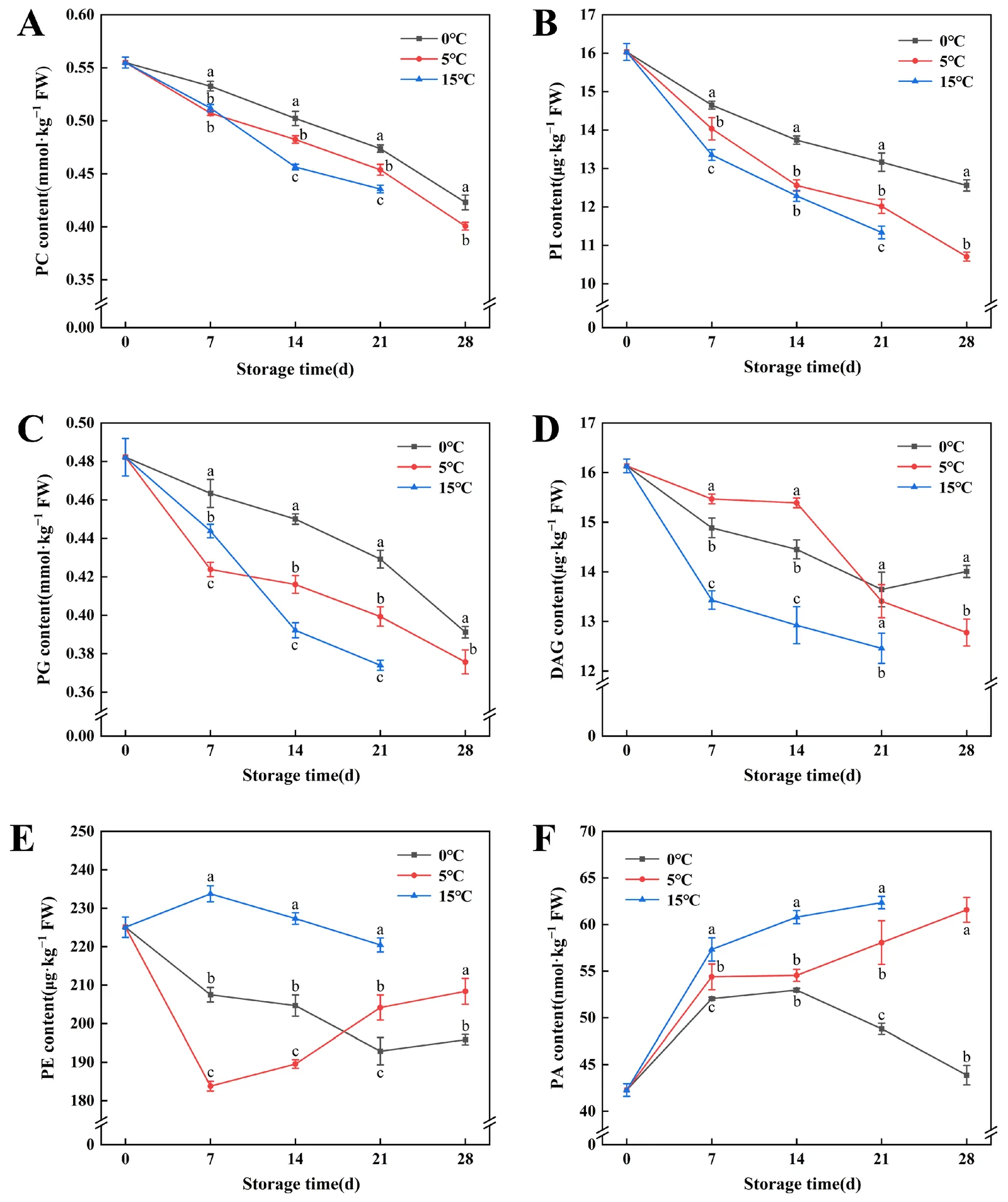
Effect of three different treatments on PC (**A**), PI (**B**), PG (**C**), DAG (**D**), PE (**E**), and PA (**F**) in peach fruit. Data are presented as mean ± standard deviation. Different letters mean statistically significant difference (*p* < 0.05) among peaches stored at the three temperatures on the same day.

The contents of PC, PI, PG, and DAG in peach fruit exhibited a gradual overall decline across all treatment groups with prolonged storage duration. The 0 °C group showed the slightest decrease in PC, PI, and PG, with levels remaining significantly higher than those in the 5 °C and 15 °C groups throughout most of the storage period. The 5 °C group maintained the highest DAG content until day 21 of storage, whereas the 15 °C group consistently exhibited the lowest DAG levels (Figure 2A–D). In addition, PE content in the 0 °C group gradually declined over time, while the 5 °C group showed a sharp initial decrease followed by a progressive increase, reaching levels significantly higher than those in the 0 °C group after 21 d. In the 15 °C group, PE content followed a trajectory of initial increase followed by decline, with the highest levels observed at 21 d of storage (Figure 2E). In the 5 °C and 15 °C groups, PA levels showed an upward trend after 7 d of storage, with the 15 °C group maintaining significantly higher levels than the 5 °C group. In contrast, PA content in the 0 °C group initially increased and then decreased, remaining consistently lower than that in the 5 °C group throughout the later storage period. By day 21, the PA content in the 0 °C group was 18.94% lower than that in the 5 °C group (Figure 2F).

### 3.3. Effects of Low-Temperature Stress on Enzyme Activities Related to Membrane Lipid Metabolism

**Figure 3:**
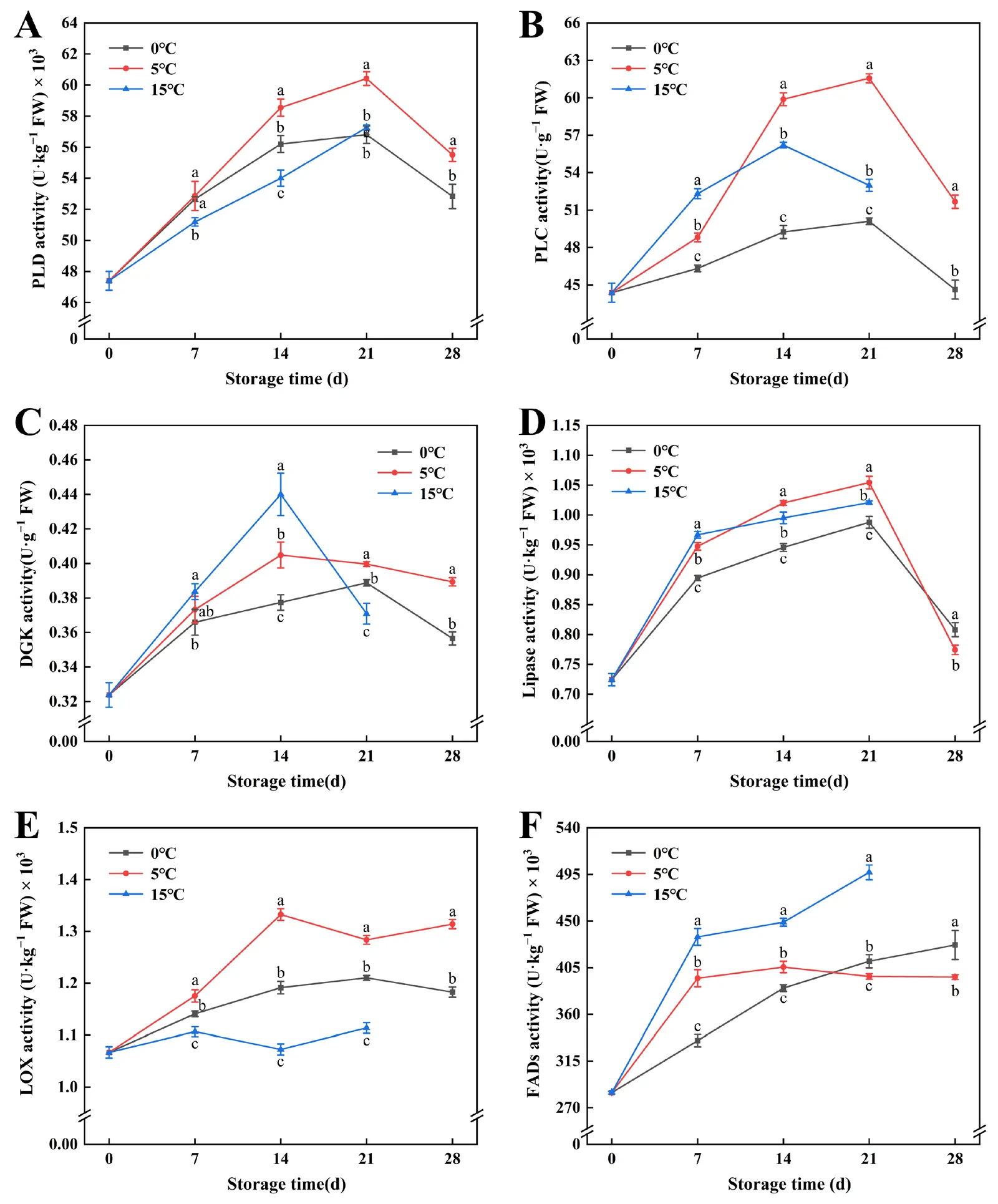
Effect of three different treatments on PLD (**A**), PLC (**B**), DGK (**C**), Lipase (**D**), LOX (**E**) and FADs (**F**) in peach fruit. Data are presented as mean ± standard deviation. Different letters mean statistically significant difference (*p* < 0.05) among peaches stored at the three temperatures on the same day.

After 7 d of storage, PLD and PLC activities in all three treatment groups exhibited an initial increase followed by a decline (Figure 3A,B). In contrast to the early storage phase, both PLD and PLC activities in the 5 °C group significantly exceeded those in the other two groups after 7 d, peaking at 21 d. DGK activity generally increased after 7 d of storage, with the highest levels observed in the 15 °C group and the lowest in the 0 °C group (Figure 3C). LOX activity in peach fruit under low-temperature storage showed a trend of initial increase followed by decrease, with the 5 °C group maintaining significantly higher activity than the 0 °C group throughout the later storage period. At day 14 of storage, lipase and LOX activities in the 5 °C group were 7.87% and 11.85% higher, respectively, than those in the 0 °C group (Figure 3D,E). In the 0 °C, 5 °C and 15 °C groups, the activity of FADs showed an overall upward trend, and the activity of the 15 °C group was always the highest. Before day 14, FADs activity in the 0 °C group was significantly lower than that in the 5 °C group; however, after day 14, activity in the 0 °C group surpassed that in the 5 °C group and remained significantly higher thereafter (Figure 3F).

### 3.4. Lipidomics Analysis of Peach Fruit Samples at 12 h

**Figure 4:**
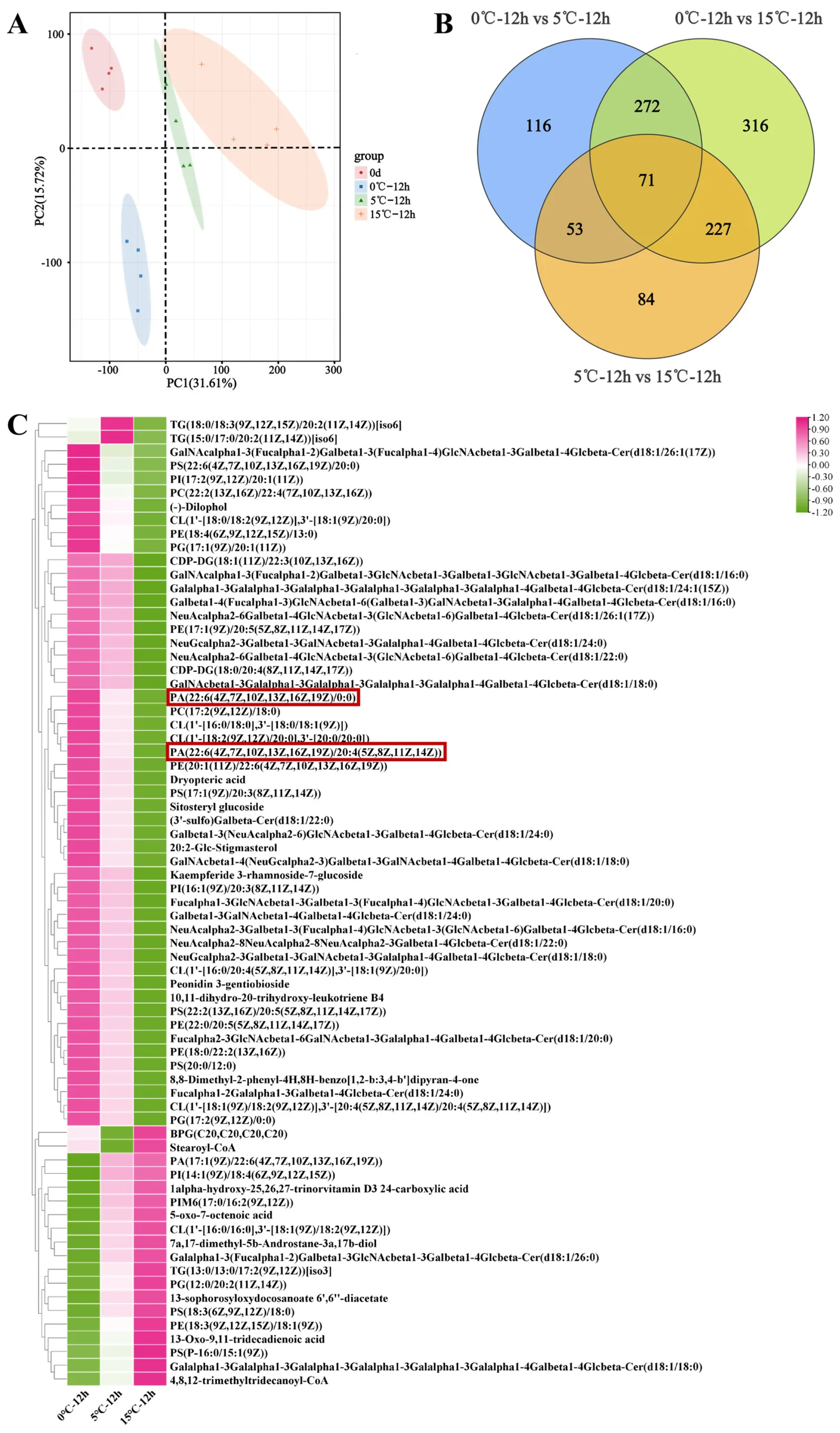
Lipidomics analysis of peach fruit samples stored at different temperatures. (**A**) PCA of samples at 0 d and after 12 h of storage at three temperatures. (**B**) Number of overlapping differentially abundant lipids identified by Venn diagram analysis among the three temperature treatment groups. (**C**) Heatmap of 71 differentially abundant lipid species in peach fruit stored at different temperatures for 12 h.

Lipidomics analysis enables comprehensive characterization of changes in membrane lipid composition associated with CI in fruits and vegetables. In this study, a total of 7507 lipid species were identified, encompassing eight major lipid categories, including glycerophospholipids, sphingolipids, and glycerides, etc. (Figure S1). To investigate early changes in signaling lipids in peach fruit, PCA was performed on all detected lipid species in samples collected at 12 h of storage. Samples from the 0 °C-12 h group were distinctly separated from those of other treatment groups, with the first principal component (PC1) explaining 31.61% of the total variance. This result indicates that cold stress induced significant alterations in the lipid profile of peach fruit within a short-term exposure (Figure 4A). Venn diagram analysis revealed 71 significantly differentially abundant lipid metabolites commonly detected across pairwise comparisons among the three temperature treatments at hourly sampling points (Figure 4B,C). Notably, PA (22:6(4Z,7Z,10Z,13Z,16Z,19Z)/0:0) and PA (22:6(4Z,7Z,10Z,13Z,16Z,19Z)/20:4(5Z,8Z,11Z,14Z)) at 0 °C were significantly higher than that at 5 °C and 15 °C (Figure 4C).

### 3.5. Lipidomics Analysis of Peach Fruit Samples at 14 d

**Figure 5:**
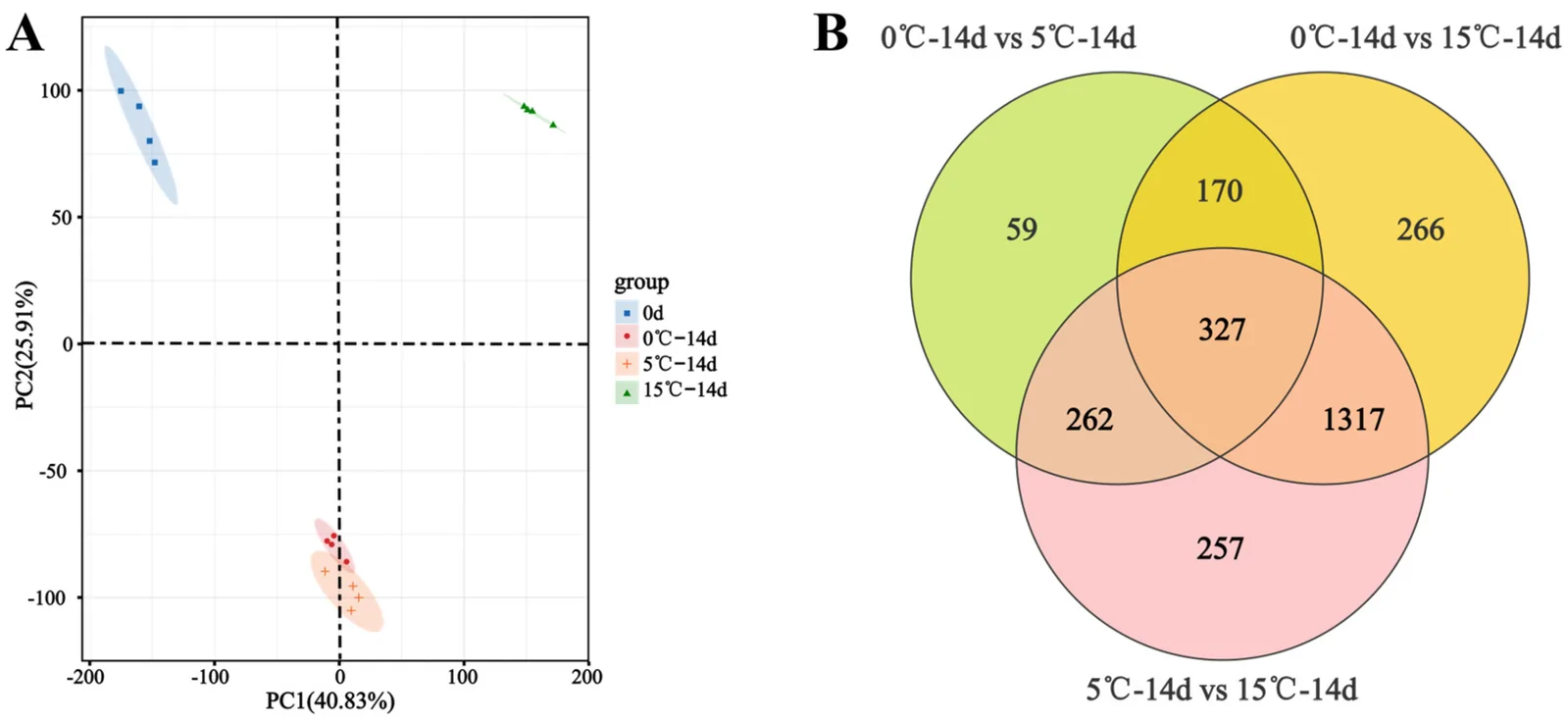
Lipidomics analysis of peach fruit samples stored at different temperatures. (**A**) PCA of samples at day 0 and after 14 d of storage at three temperatures. (**B**) Number of overlapping differentially abundant lipids identified by Venn diagram analysis among the three temperature treatment groups.

To investigate identify key lipid components associated with CI in peach fruit, PCA was performed on all detected lipid species in samples collected 14 d of storage. Samples from the 0 °C-14 d and 5 °C-14 d treatment groups were distinctly separated from those of the other two groups, with PC1 accounting for 40.83% of the total variance. This finding suggests that CI significantly alters the lipid composition of peach fruit. Notably, although the 0 °C-14 d and 5 °C-14 d groups were not completely separated, they exhibited minimal overlap, indicating distinct lipid profiles between these two temperature treatments (Figure 5A). Venn diagram analysis identified 327 significantly differentially abundant lipid metabolites commonly detected across pairwise comparisons among the three temperature treatments at weekly sampling points (Figure 5B). Among these 327 significantly differentially abundant lipid metabolites, glycerophospholipids represented the most abundant category, comprising 112 species, followed by sphingolipids, fatty acids, glycerides, and others (Figure S2).

### 3.6. Identification of Key Lipid Species Associated with Chilling Injury

**Figure 6:**
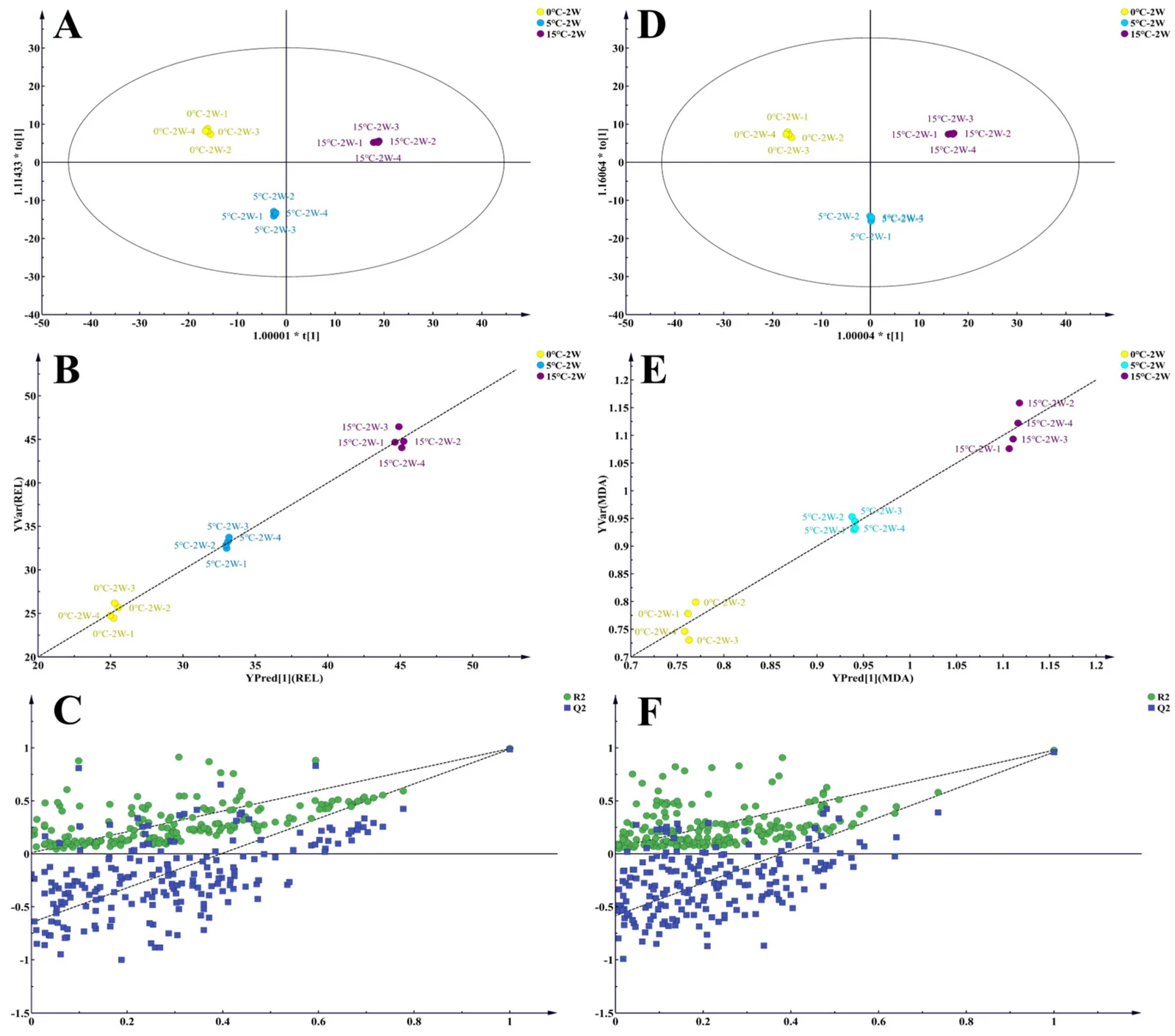
Correlation analysis and model validation between CI indicators and lipidomic profiles. (**A**) OPLS score plot of the relative REL. (**B**) Observed vs. predicted validation plot for the REL model. (**C**) Permutation test results for the REL model. (**D**) OPLS score plot of the MDA content model. (**E**) Observed vs. predicted validation plot for the MDA content model. (**F**) Permutation test results for the MDA content model. Different letters mean statistically significant difference (*p* < 0.05) among peaches stored at the three temperatures on the same day.

To investigate the relationship between lipid metabolic changes and CI in peach fruit, OPLS regression analysis was conducted between the 327 differentially abundant lipids identified at 0 °C-14 d, 5 °C-14 d, and 15 °C-14 d and two key physiological indicators, namely REL and MDA content. Model validity was assessed using observed vs. predicted value plots and permutation tests. The OPLS score plots revealed distinct separation among the 0 °C-14 d, 5 °C-14 d, and 15 °C-14 d treatment groups (Figure 6A,D). The model constructed for REL exhibited high explanatory and predictive power, with R^2^ = 0.992 and Q^2^ = 0.988. Similarly, the model for MDA content yielded R^2^ = 0.977 and Q^2^ = 0.960, indicating strong correlations between CI indicators and differential lipid profiles. Validation of the REL model using observed vs. predicted values produced a coefficient of determination R^2^ of 0.9925 (Figure 6B), while permutation testing resulted in R^2^ = 0.0586 and Q^2^ = −0.649 (Figure 6C). For the MDA content model, observed vs. predicted validation gave an R^2^ of 0.9771 (Figure 6E), and permutation testing yielded R^2^ = 0.0586 and Q^2^ = −0.585 (Figure 6F), confirming the absence of overfitting in both models. Based on the established models, key differential lipids strongly correlated with CI were identified using variable importance in projection (VIP) scores. Lipids with the highest VIP values (top 30) were selected, along with those exhibiting regression coefficients exceeding 0.97. In the REL model, the 30 lipids with the highest VIP values comprised 10 sphingolipids, 6 glycerophospholipids, 6 fatty acids, 4 glycerides, and 4 other minor lipid species (Table 1). In the MDA content model, the top 30 lipids by VIP score included 13 glycerophospholipids, 6 sphingolipids, 4 fatty acids, 2 glycerides, and 5 other minor lipid species (Table 2). Comparative analysis of the key differential lipids identified by both models revealed nine overlapping lipid species, including 3 sphingolipids, 2 glycerophospholipids, 2 glycerides, and 1 fatty acid. Given that ceramides are associated with apoptosis and stress signaling, while ether lipids are implicated in membrane stability and antioxidant functions, the following lipids were identified as key differentially abundant metabolites associated with CI in peach fruit: Cer (d16:1/24:0), NeuAα2-3Galβ-Cer (d18:1/22:0), Fucα1-2Galβ1-3 (Fucα1-4) GlcNAcβ1-3Galβ1-4Glcβ-Cer (d18:1/16:0), PE (O-16:0/19:1(9Z)), and PA (O-18:0/14:0).

## 4. Discussion

Low-temperature storage is the most widely adopted strategy for extending the postharvest shelf life of horticultural fruits, as it effectively suppresses cellular metabolism and preserves fruit quality [36]. However, peach fruit is highly sensitive to low temperatures, and exposure to cold stress induces a series of physiological perturbations [37]. In this study, CI symptoms were most pronounced in peach stored at 5 °C. IB was first observed after 14 d of storage and continued to worsen, reaching a CI index of 0.87 at 28 d, which was 2.56 times higher than the 0.34 recorded at 14 d. This aggravation of CI was accompanied by elevated REL and MDA content—established indicators of membrane lipid peroxidation and loss of membrane integrity [35]. By 21 d, REL in the 5 °C group reached 46.29%, which was 45.52% higher than that in the 0 °C group (31.81%), while MDA content in the 5 °C group reached 1.07 μmol g^−1^ FW, representing a 28.92% increase compared with the 0 °C group (0.83 μmol g^−1^ FW). These findings suggest that storage at 5 °C induces substantial oxidative damage to cellular membranes, constituting a primary factor underlying CI development. In contrast, storage at 0 °C significantly attenuated these physiological changes, preserved membrane integrity, and minimized the severity of CI. This observation is consistent with the widely accepted view that membrane dysfunction represents the initial event in CI development [16]. At 5 °C, low-temperature stress likely triggers a phase transition in membrane lipids, leading to increased membrane permeability and the generation of ROS, while accumulated MDA exacerbates oxidative damage to membrane lipids. Storage at 0 °C effectively interrupted this vicious cycle, thereby maintaining membrane structural and functional integrity. Notably, although storage at 15 °C prevented the onset of CI, it markedly accelerated fruit senescence. This was reflected by a sharp increase in the decay index over time, reaching 0.58 at 21 d and 1.0 at 28 d of storage. Elevated REL and MDA levels were also observed throughout the storage period. This phenomenon is likely attributable to metabolic dysregulation associated with accelerated aging at this temperature.

The cell membrane is regarded as the initial site of CI, and its integrity and fluidity serve as hallmark indicators of the severity of CI [38,39]. In the present study, with prolonged storage duration, the contents of PC, PI, PG and DAG in all treatment groups collectively exhibited a gradual declining trend. Notably, the contents of PC, PI, and PG in the 0 °C group exhibited the slowest decline and remained significantly higher than those in the 5 °C and 15 °C groups for the majority of the storage period. At 14 d of storage, the PC content in the 0 °C group was 4.35% higher than that in the 5 °C group, while the PI and PG contents were 9.40% and 8.17% higher, respectively. These results indicate that storage at 0 °C more effectively retards the degradation of structural membrane phospholipids and preserves membrane skeleton integrity. This phenomenon is consistent with the findings of Song et al. in peach fruit, who demonstrated that storage at 0 °C delayed the upregulation of membrane lipid metabolism-related genes and the process of phospholipid degradation, thereby maintaining higher levels of PC and PE and postponing the occurrence of CI [31]. In this study, after 14 d of storage, fruit stored at 0 °C showed significantly higher FADs enzyme activity than those stored at 5 °C, with a 7.83% increase. In contrast, lipase and LOX activities at 0 °C were significantly lower, exhibiting reductions of 7.87% and 11.85%, respectively, relative to the 5 °C group. This result is consistent with previous studies. It has been reported that peach fruit stored at 0 °C exhibits higher membrane lipid unsaturation and membrane fluidity, along with elevated expression of ω-3 fatty acid desaturase genes and higher levels of C18:3, compared to those stored at 5 °C. That study also observed a weaker ability to maintain membrane lipid unsaturation at 5 °C [8]. These findings indicate that the essence of effective cold adaptation lies not merely in the intensity of the initial stress response, but rather in the ability to establish and maintain a synergistic balance between membrane phospholipid composition and unsaturation. Storage at 0 °C preserves membrane homeostasis by retarding phospholipid degradation and sustaining FADs, lipase, and LOX-mediated unsaturation, thereby alleviating CI in peach fruit. In contrast, the rapid degradation of structural phospholipids and the sustained decline in membrane fluidity at 5 °C ultimately exacerbate CI. However, although the above results reveal the importance of the synergistic maintenance of membrane lipid composition and unsaturation in preserving membrane homeostasis, how differential temperatures regulate peach fruit cold adaptation through membrane lipid signaling pathways remains to be elucidated.

In the present study, significant lipidome remodeling was observed within the first 12 h of cold stress, particularly at 0 °C. Principal component analysis revealed a clear separation between the 0 °C-12 h sample and those from the 5 °C-12 h and 15 °C-12 h groups, accompanied by the rapid accumulation of multiple signaling lipids, including PA, under 0 °C conditions. This finding indicates that peach fruit rapidly perceives low-temperature signals at the cellular level and initiates adaptive responses through the activation of membrane lipid metabolic networks. As a key second messenger, the rapid and transient accumulation of PA is considered an early event in plant low-temperature signal transduction [40,41]. Studies have shown that PA, as a signaling phospholipid, plays a central role in plant responses to cold stress by binding to target proteins to enhance cold response and adaptation [25]. A pioneering study in *Arabidopsis thaliana* suspension cells demonstrated for the first time that cold treatment simultaneously activates PLC and PLD, leading to rapid PA accumulation in a process dependent on Ca^2+^ influx. This finding provided direct cytological evidence for the early PA response in plant cold signal transduction [42]. Lipidomic analysis conducted on day 14 of storage revealed distinct separation among the 0 °C-14 d, 5 °C-14 d, and 15 °C-14 d samples in principal component analysis, indicating that CI induced substantial alterations in membrane lipid composition. Correlation analysis between differentially abundant lipids and CI parameters further demonstrated that PA accumulated markedly during the later storage stage, emerging as a significant contributor to the differential severity of CI observed among temperature treatments. Correlation analysis indicated that PA content was significantly positively correlated with REL and MDA content, while showing a negative correlation with the degree of membrane lipid unsaturation. These findings confirm that PA accumulation during the later stage is a key factor driving the loss of membrane integrity and the exacerbation of CI. Similarly, Song et al. found that in peach fruit stored at 4 °C, PA accumulated significantly during the later storage stage, accompanied by the upregulation of genes involved in membrane lipid metabolism, thereby inducing CI. However, that study did not note the signaling role of PA during the early stage. Notably, PA (22:6(4Z,7Z,10Z,13Z,16Z,19Z)/0:0) and PA (22:6(4Z,7Z,10Z,13Z,16Z,19Z)/20:4(5Z,8Z,11Z,14Z)) levels in fruit stored at 0 °C exhibited a transient surge at 12 h, whereas the PA content in fruit stored at 5 °C increased steadily after 7 d of storage, reaching a level 1.19-fold higher than that in the 0 °C group by 21 d. This temporal pattern highlights the dual role of PA in plant stress responses: controlled and transient PA accumulation is essential for initiating cold signal transduction and adaptive responses, whereas excessive and sustained PA accumulation may exacerbate membrane damage by promoting lipid peroxidation and disrupting bilayer stability. This interpretation is consistent with recent findings on PA dynamics in other chilling-sensitive fruits, such as banana and pineapple [10,43].

Although numerous studies have elucidated the functions of PLD, novel roles of PLDs and their product PA are still being discovered. In rice, a 2D-DIGE proteomic study revealed that 26 proteins changed within 5 min of chilling stress, with OsPLDα1 upregulated as early as 1 min—making it one of the most rapid cold-responsive proteins identified. OsPLDα1 hydrolyzes phosphatidylcholine to produce PA, which directly binds to OsMPK6 and OsSIZ1, thereby regulating the expression of OsDREB1 transcription factors. Loss-of-function mutants exhibit increased sensitivity to chilling and freezing stress. Furthermore, OsPLDα1 expression is transcriptionally regulated by OsDREB1A, forming a positive feedback loop (PLD-PA-DREB1) [44]. This finding directly demonstrates that a single PLD gene can significantly enhance stress tolerance, supporting our hypothesis that the PpPLD family, particularly members containing specific stress-responsive promoter elements, may play key roles in peach under cold stress. In plants, PLD and PA have been demonstrated to play central roles in responding to hormonal and environmental stimuli. For example, the plant hormone abscisic acid has been shown to activate PLDα1 to produce PA, thereby regulating stomatal closure [45]. These studies collectively underscore that the PLD-PA module is a conserved signaling hub integrating diverse stress signals. Furthermore, further elucidating the interactions between the PLD-PA module and the MAPK cascade as well as Ca^2+^ signaling pathways will contribute to the construction of an integrative signaling regulatory network, enabling systematic dissection of the complex mechanisms underlying postharvest low-temperature adaptation in peach fruit at the molecular level.

A range of postharvest pretreatments have been developed to alleviate CI in peach fruit. Most of these strategies stabilize membrane integrity, modulate phospholipase activity, and reprogram lipid metabolism to counteract cold-induced damage. Many effective pretreatments play a protective role by regulating PA accumulation and downstream membrane lipid remodeling, which is consistent with our current observations. Notably, these pretreatments can be broadly categorized into two groups based on their regulatory direction of PA. The first group features the suppression of PA accumulation, with representative strategies including 24-epibrassinolide (EBR), cold shock (CS), and γ-aminobutyric acid (GABA) treatments. EBR suppresses the activities and expression of PLD, LOX, and lipase, thereby reducing the hydrolysis of PC and PI to PA and maintaining membrane stability [46]. CS similarly inhibits the activities and expression of membrane lipid-degrading enzymes, maintaining higher fatty acid unsaturation and lower PA content [47]. GABA attenuates PA biosynthesis and maintains higher PC and PE levels to enhance cold tolerance [48]. The common logic underlying these strategies is that excessive PA accumulation under low-temperature stress is itself a cause of membrane damage; therefore, blocking the overproduction of PA effectively alleviates CI—a finding entirely consistent with our observation of high PA accumulation corresponding to severe CI during the later stage of 5 °C storage. The second group places greater emphasis on exploiting the signaling function of PA. Methyl jasmonate (MeJA) treatment alleviates CI by promoting phospholipid remodeling and jasmonic acid signaling [49], with its mechanism likely involving early transient PA accumulation to trigger downstream defense responses. β-Ionone mitigates CI by modulating key membrane lipid components, including PA, and inhibiting degradative enzyme activities [50]. These strategies suggest that PA is not always a deleterious metabolic intermediate—within an appropriate temporal window, controlled PA accumulation is essential for activating cold acclimation signaling. This view is fully consistent with the transient PA burst observed in our study at 12 h of 0 °C storage, which serves as a “trigger switch” for initiating cold adaptation. Therefore, whether inhibitory or activating, these strategies ultimately point to the same core conclusion: the dynamic balance of PA is more important than its absolute level. When PA transiently rises at the right time and with the right magnitude, it activates protective signals; when PA accumulates persistently at high levels, it exacerbates membrane damage. This is precisely the case at 5 °C storage—lagging and sustained PA accumulation leads to aggravated membrane damage—whereas at 0 °C, PA achieves an optimal balance between signaling and protection through a transient burst followed by rapid decline. These pretreatment strategies, viewed from different angles, validate this model and provide theoretical foundations and potential targets for future precision regulation strategies targeting PA dynamic balance.

## 5. Conclusions

Based on these findings, we propose a comprehensive model for the alleviation of CI in peach fruit stored at 0 °C, with PA as the central hub. Compared with storage at 5 °C and 15 °C, peach fruit stored at 0 °C maintained significantly higher levels of structural phospholipids, indicating that lipid remodeling under this temperature condition contributes to enhancing the structural stability of cell membranes, thereby alleviating CI. Additionally, storage at 0 °C maintained higher FAD activity while significantly suppressing lipase and LOX activities. This coordinated regulation favors the preservation of membrane lipid unsaturation and fluidity, thus mitigating membrane damage caused by CI. Critically, lipidomics analysis revealed that PA at 0 °C exhibited a pronounced transient accumulation at the early stage of storage (12 h), followed by maintenance at a relatively low level throughout the later storage period. This dynamic pattern allows for the initiation of cold acclimation signaling while avoiding membrane damage that could result from sustained PA accumulation. The above mechanisms collectively establish sustainable membrane lipid homeostasis, conferring enhanced low-temperature adaptability to the fruit. In contrast, under 5 °C storage, excessive and persistent PA accumulation, together with intensified lipid peroxidation, aggravates CI. Notably, storage at 15 °C represents a non-low-temperature stress condition, under which fruit ripen normally without exhibiting CI symptoms. Under this condition, PA accumulation and membrane lipid remodeling remained at basal levels, further confirming that the pronounced lipid signaling responses observed at 0 °C and 5 °C were specifically induced by cold stress, rather than being a general concomitant of the ripening process. However, although 15 °C can fully prevent CI, its negative effects of accelerating fruit ripening and shortening storage life render it unsuitable for long-term preservation. Taken together, this model reveals that the differential dynamics of PA and membrane lipid remodeling across storage temperatures are key determinants of whether CI occurs and its severity, offering new insights into the mechanisms by which temperature modulates postharvest cold tolerance in fruit and providing a theoretical foundation for the development of precision preservation strategies in the peach cold chain. This sets the foundation for maintaining membrane integrity and alleviating CI throughout the entire low-temperature storage period. This model provides new insights into the mechanisms by which temperature modulates postharvest fruit cold tolerance and offers a theoretical basis for the development of precision preservation strategies in the peach fruit cold chain.

## Figures and Tables

**Table 1:** Identification of key differential lipids in the relative electrolyte leakage model.

Differential Lipids	VIP	Coefficient
Cer(d16:1/24:0)	1.215	0.997
1-(10-methyl-hexadecanoyl-2-(8-[3]-ladderane-octanyl)-sn-glycerol	1.213	0.995
PA(O-18:0/14:0)	1.213	0.995
19-methyl-5E,9E-eicosadienoic acid	1.213	0.995
Fucalpha1-2Galbeta1-3(Fucalpha1-4)GlcNAcbeta1-3Galbeta1-4Glcbeta-Cer(d18:1/16:0)	1.211	0.994
19Z-pentacosenoic acid	1.211	0.994
PE(P-18:0/19:0)	1.210	0.992
PE(O-16:0/19:1(9Z))	1.209	0.992
PA(15:0/18:2(9Z,12Z))	1.206	0.989
NeuAcalpha2-3Galbeta-Cer(d18:1/22:0)	1.206	0.989
TG(14:0/18:2(9Z,12Z)/18:4(6Z,9Z,12Z,15Z))[iso6]	1.205	0.988
Calditoglycerocaldachaeol	1.205	0.988
Cer(t18:0/16:0(2OH))	1.203	0.987
Fucalpha2-3(Galbeta1-4)GlcNAcbeta1-6GalNAcbeta1-3Galalpha1-4Galbeta1-4Glcbeta-Cer(d18:1/16:0)	1.203	0.987
Galbeta1-3(Fucalpha1-4)GlcNAcbeta1-3Galbeta1-3GlcNAcbeta1-3Galbeta1-4Glcbeta-Cer(d18:1/16:0)	1.203	0.986
bayogenin 3-O-cellobioside	1.203	−0.986
PA(18:1(9Z)/16:0)	1.202	−0.986
GalNAcalpha1-3Galbeta1-3GlcNAcbeta1-3Galbeta1-3GlcNAcbeta1-3Galbeta1-4Glcbeta-Cer(d18:1/26:0)	1.202	0.986
PGF1beta	1.200	0.984
TG(12:0/12:0/17:0)[iso3]	1.199	0.984
18-nonadecynoic acid	1.199	0.984
EI-1625-2	1.197	−0.982
PC(O-20:0/22:4(7Z,10Z,13Z,16Z))	1.197	0.982
9-OAc-NeuAcalpha2-3Galbeta1-3GalNAcbeta1-4(NeuAcalpha2-3)Galbeta1-4Glcbeta-Cer(d18:1/24:1(15Z))	1.196	−0.981
zymosterol intermediate 1b	1.196	0.981
Castasterone	1.195	0.981
3-hydroxy-5-methoxy-6-prenylstilbene-2-carboxylic acid	1.192	0.978
Galbeta1-3(GlcNAcbeta1-6)GalNAcbeta1-3Galalpha1-4Galbeta1-4Glcbeta-Cer(d18:1/20:0)	1.191	0.977
9-OAc-NeuAcalpha2-8NeuAcalpha2-8NeuAcalpha2-3Galbeta1-4Glcbeta-Cer(d18:1/16:0)	1.191	−0.977
24-hydroxy-tetracosanoic acid	1.191	0.977

**Table 2:** Identification of key differential lipids in the malondialdehyde content model.

Differential Lipids	VIP	Coefficient
PA(18:1(9Z)/16:0)	1.271	−0.998
bayogenin 3-O-cellobioside	1.268	−0.996
EI-1625-2	1.265	−0.994
9-OAc-NeuAcalpha2-8NeuAcalpha2-8NeuAcalpha2-3Galbeta1-4Glcbeta-Cer(d18:1/16:0)	1.263	−0.992
PG(20:2(11Z,14Z)/18:1(9Z))	1.262	−0.992
19Z-pentacosenoic acid	1.261	0.991
9-OAc-NeuAcalpha2-3Galbeta1-3GalNAcbeta1-4(NeuAcalpha2-3)Galbeta1-4Glcbeta-Cer(d18:1/24:1(15Z))	1.261	−0.991
PI(20:2(11Z,14Z)/18:0)	1.260	−0.990
PE(O-16:0/19:1(9Z))	1.259	0.989
Vitexin 2’’-O-rhamnoside-4’’’-acetate	1.259	−0.989
CL(1’-[16:0/20:4(5Z,8Z,11Z,14Z)],3’-[20:4(5Z,8Z,11Z,14Z)/20:4(5Z,8Z,11Z,14Z)])	1.258	−0.988
Ulexin C	1.258	−0.988
Eriosemaone C	1.257	−0.988
Delphinidin 3-(2G-xylosylrutinoside)	1.256	−0.987
PG(17:1(9Z)/22:6(4Z,7Z,10Z,13Z,16Z,19Z))	1.255	−0.986
CL(1’-[16:0/18:1(9Z)],3’-[18:0/20:4(5Z,8Z,11Z,14Z)])	1.252	−0.984
PS(17:2(9Z,12Z)/22:6(4Z,7Z,10Z,13Z,16Z,19Z))	1.251	−0.983
NeuAcalpha2-8NeuAcalpha2-8NeuAcalpha2-3Galbeta1-4Glcbeta-Cer(d18:1/22:0)	1.251	−0.983
PA(12:0/21:0)	1.251	−0.983
Cer(d16:1/24:0)	1.251	0.983
PC(18:4(6Z,9Z,12Z,15Z)/22:6(4Z,7Z,10Z,13Z,16Z,19Z))	1.248	−0.981
PA(O-18:0/14:0)	1.247	0.980
1-(10-methyl-hexadecanoyl-2-(8-[3]-ladderane-octanyl)-sn-glycerol	1.247	0.980
19-methyl-5E,9E-eicosadienoic acid	1.246	0.979
Calditoglycerocaldachaeol	1.245	0.978
PE(20:1(11Z)/22:6(4Z,7Z,10Z,13Z,16Z,19Z))	1.245	−0.978
NeuAcalpha2-3Galbeta-Cer(d18:1/22:0)	1.244	0.978
Fucalpha1-2Galbeta1-3(Fucalpha1-4)GlcNAcbeta1-3Galbeta1-4Glcbeta-Cer(d18:1/16:0)	1.243	0.977
CL(1’-[16:0/18:1(9Z)],3’-[18:0/18:1(9Z)])	1.243	−0.977
Docosanediol-1,14-disulfate	1.242	−0.976

## Data Availability

The original contributions presented in this study are included in the article/supplementary material. Further inquiries can be directed to the corresponding authors.

## Supplementary Materials

The following supporting information can be downloaded at https://www.mdpi.com/article/10.3390/foods15162810/s1: Figure S1: Composition and proportions of the eight major lipid categories. Figure S2: Heatmap of common differentially abundant lipid species in peach fruit stored at different temperatures for 14 days. (A) Glycerophospholipids. (B) Glycerolipids. (C) Sphingolipids. (D) Fatty acyls. (E) Polyketides. (F) Sterol lipids. (G) Prenol lipids.
