# Supplementary material for: Phosphatidic Acid Serves as an Early Signaling Molecule Involved in Membrane Lipid Metabolism to Alleviate Postharvest Chilling Injury in Peach Fruit
Source: Foods. 2026 Aug 12;15(16):2810. doi: 10.3390/foods15162810 (PMC13511788; doi:10.3390/foods15162810)
Supplement: Supplementary file 1 [file foods-15-02810-s001.zip › foods-4428153-supplementary.pdf]

# Phosphatidic acid serves as an early signaling molecule involved in membrane lipid metabolism to alleviate postharvest chilling injury in peach fruit

Yun Zhang, Fengyuan Xie, Ziyi Wang, Yonghua Zheng, Liangyi Zhao*, Peng Jin*

College of Food Science and Technology, Nanjing Agricultural University, Nanjing, 210095, China

*Corresponding authors:

E-mail address: t2024063@njau.edu.cn (L. Zhao), pjin@njau.edu.cn (P. Jin)

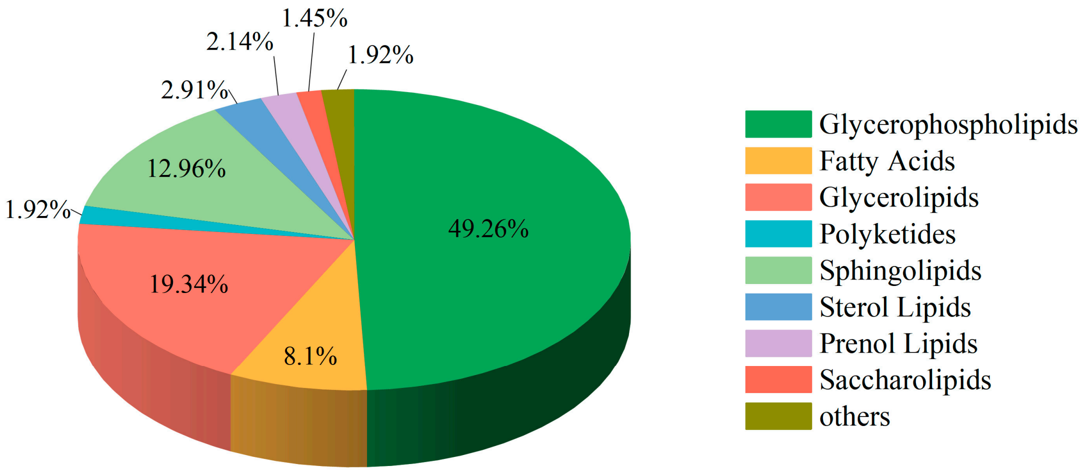


Figure S1 Composition and proportions of the eight major lipid categories

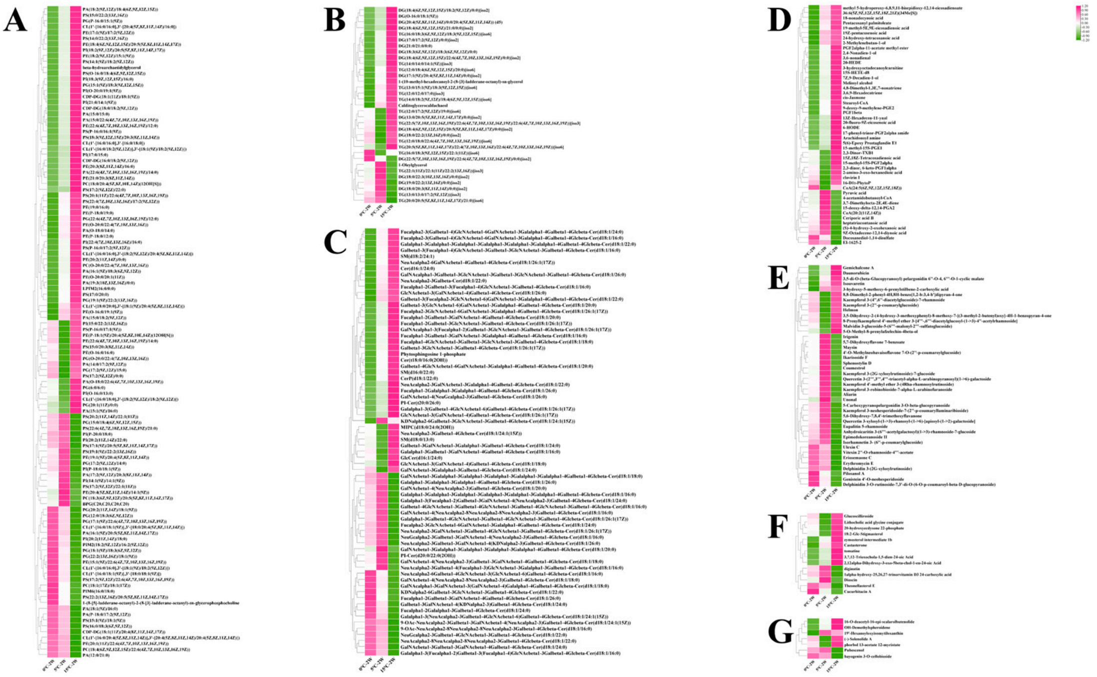


Figure S2 Heatmap of common differentially abundant lipid species in peach fruit stored at different temperatures for 14 days. (A) Glycerophospholipids. (B) Glycerolipids. (C) Sphingolipids. (D) Fatty acyls. (E) Polyketides. (F) Sterol lipids. (G) Prenol lipids.
